# Kimchi Modulates Epithelial–Macrophage Responses in a Caco-2/THP-1 Co-Culture Model of Inflammatory Lipid Stress

**DOI:** 10.4014/jmb.2608.08002

**Published:** 2026-10-15

**Authors:** Ha-Rin Moon, Jieun Lee, Min-Sung Kwon, Wooje Lee

**Affiliations:** Global Convergence Research Division, World Institute of Kimchi, 86 Kimchi-ro, Nam-gu, Gwangju 61755, Republic of Korea

**Keywords:** Kimchi, Intestinal epithelium, Macrophage, Inflammatory lipid stress, Lipid accumulation

## Abstract

Kimchi is a traditional fermented vegetable food with potential benefits for intestinal and metabolic health. However, its effects on intestinal epithelial barrier function and macrophage inflammatory responses and lipid accumulation under inflammatory lipid stress remain unclear. We used a compartmentalized Caco-2/THP-1 Transwell co-culture model to evaluate the effects of filtered preparations of spontaneously fermented kimchi (S-K) and *Leuconostoc mesenteroides* KCKM0828 starter-fermented kimchi (LMS-K). Differentiated Caco-2 monolayers were cultured above THP-1-derived macrophages. S-K or LMS-K was applied to the apical compartment, while the basolateral compartment was challenged with LPS and ox-LDL for 24 h. Cellular responses in both compartments were evaluated using gene and protein expression analyses, immunofluorescence, FITC-dextran permeability assays, and Oil Red O staining. Under LPS/ox-LDL challenge, both S-K and LMS-K attenuated epithelial inflammatory responses, reduced FITC-dextran passage, and increased mean E-cadherin fluorescence intensity compared with the challenged vehicle control, whereas only LMS-K significantly increased *TJP1* mRNA expression. In basolateral macrophages, apical treatment with either preparation was associated with lower inflammatory marker expression and lipid accumulation, higher PPARα protein abundance and LXRα/β immunoreactivity, and lower CD36 and LOX-1 expression. Overall, apical treatment with either kimchi preparation attenuated epithelial barrier-associated disruption, alongside reductions in macrophage inflammatory markers and lipid accumulation under LPS/ox-LDL challenge. These findings indicate that apical kimchi treatment modulates epithelial and macrophage responses within the compartmentalized co-culture model under combined inflammatory and lipid stress.

## Introduction

Kimchi is a traditional Korean fermented vegetable food. Its fermentation is largely driven by lactic acid bacteria, resulting in characteristic microbial communities and metabolite profiles [1]. Previous studies have reported that kimchi and kimchi-derived components can attenuate inflammatory and metabolic disturbances, alter gut microbial composition, and support intestinal barrier-associated functions in cellular and animal models [2-4]. Clinical studies have suggested diverse benefits of kimchi consumption, including metabolic and gastrointestinal outcomes as well as systemic immune modulation [5-7]. However, whether kimchi can simultaneously modulate intestinal epithelial barrier function, macrophage inflammatory responses, and lipid accumulation remains unclear.

The intestinal epithelium is the first host interface encountered by ingested foods and fermentation-derived components [8, 9]. It forms a selectively permeable barrier that permits the transport of nutrients and metabolites while restricting the passage of potentially harmful luminal substances [8, 10]. Disruption of this barrier increases intestinal permeability and can contribute to mucosal inflammation [11]. Epithelial barrier integrity is therefore an important outcome when evaluating the intestinal effects of fermented foods [10, 12].

Beyond its physical barrier role, the intestinal epithelium serves as a regulatory interface between luminal dietary and microbial factors and immune cells in the underlying mucosa [13, 14]. Epithelial cells integrate luminal signals to regulate inflammatory pathways, junctional organization, barrier permeability, and the release of soluble mediators [13-15]. In turn, signals from mucosal immune cells can modify epithelial inflammatory and barrier responses [15, 16]. This reciprocal relationship links epithelial integrity with the regulation of mucosal inflammatory responses [13, 14].

Macrophages are key components of the epithelial–immune network [17, 18]. Intestinal macrophages support mucosal homeostasis through microbial clearance, immune surveillance, and tissue remodeling and can contribute to epithelial repair following injury [18-20]. Conversely, sustained activation, particularly in conjunction with altered lipid metabolism, can increase inflammatory mediator production and compromise epithelial barrier integrity [14, 21-23].

During intestinal inflammation, elevated oxidative stress may create conditions that favor LDL oxidation and local ox-LDL formation [24, 25]. Scavenger receptor-mediated ox-LDL uptake can promote intracellular lipid accumulation and disturb cholesterol homeostasis in macrophages, while ox-LDL also engages inflammatory pathways in intestinal epithelial cells [23, 25, 26]. Combined stimulation with LPS and ox-LDL provides an *in vitro* approach to examining inflammatory responses under lipid-loading conditions [27, 28].

Conventional monoculture models evaluate epithelial and immune cells separately, limiting assessment of responses within a shared environment [29, 30]. Because intestinal epithelial cells constitute the primary luminal interface for sensing food- and microbe-derived signals before communicating with underlying immune cells [31], direct macrophage exposure to food preparations does not reproduce this spatial sequence. Compartmentalized co-culture models better reflect this spatial arrangement by enabling apical treatment and basolateral assessment of macrophage responses [29, 30]. This approach allows epithelial and macrophage responses to be examined in relation to one another while preserving their compartmental separation [32, 33].

Accordingly, we used a compartmentalized Caco-2/THP-1 Transwell co-culture model to evaluate responses to apical treatment with preparations of spontaneously fermented kimchi (S-K) or *Leuconostoc mesenteroides* KCKM0828 starter-fermented kimchi (LMS-K) under basolateral LPS/ox-LDL challenge. We assessed epithelial inflammation and barrier function together with macrophage inflammatory marker expression, lipid accumulation, lipid-regulatory markers, and ox-LDL uptake-related receptors.

## Materials and Methods

### Materials

Human monocytic THP-1 cells and human colorectal adenocarcinoma Caco-2 cells were obtained from the Korean Cell Line Bank (Republic of Korea). LPS (Cat. No. L4391), phorbol 12-myristate 13-acetate (PMA; Cat. No. P1585), fluorescein isothiocyanate (FITC)-dextran (4 kDa; Cat. No. 46944), and Oil Red O (Cat. No. O1391) were purchased from Sigma-Aldrich (USA). The CellTiter 96^®^ AQueous One Solution Cell Proliferation Assay (MTS; Cat. No. G3581) was purchased from Promega (USA). The bicinchoninic acid (BCA) protein assay kit (Cat. No. 23227) and ox-LDL (Cat. No. L34357) were obtained from Thermo Fisher Scientific (USA). Transwell^®^ inserts (0.4 μm pore size, polycarbonate membrane; Cat. No. 3412) were purchased from Corning Inc. (USA). Specific quantitative polymerase chain reaction (qPCR) primers for *TJP1*, *RELA*, *PPARA*, *NR1H3*, *OLR1*, *CD36*, *PTGS2*, and *GAPDH* were purchased from Bioneer (Republic of Korea). Unless otherwise specified, all other chemicals and reagents were purchased from Sigma-Aldrich.

### Preparation of Kimchi Samples

S-K and LMS-K used in this study were prepared based on previously described protocols [5, 34], with the specific preparation conditions described below. Briefly, kimchi was prepared using salted kimchi cabbage (*Brassica rapa* subsp. *pekinensis*) and a standardized seasoning mixture containing red pepper, fish sauce, garlic, ginger, radish, and onion. For LMS-K, the kimchi mixture was inoculated with *L. mesenteroides* KCKM0828 at 10^7^ CFU/mL, whereas S-K was prepared without starter inoculation and allowed to ferment spontaneously. Fermentation was performed at 6°C for 14 days. The microbial and metabolite profiles of these preparations were previously characterized, with *L. sakei* and *L. mesenteroides* identified as the dominant taxa in S-K and LMS-K, respectively [5].

After fermentation, the kimchi samples were freeze-dried, ground into powder, and dispersed in dimethyl sulfoxide (DMSO; Duchefa Biochemie, The Netherlands; Cat. No. D1370.1000) at 100 mg of powder per mL of DMSO. The preparations were filtered through a 0.22-μm syringe filter (Sartorius, Germany), and the resulting soluble fractions were stored at −20°C until use. For co-culture experiments, these fractions were diluted with culture medium to a final kimchi-equivalent concentration of 1 mg/mL, defined on the basis of the starting freeze-dried powder rather than the recovered soluble solids after filtration. The initial DMSO concentration in the apical treatment medium was 1% (v/v). The corresponding co-culture control groups, including the unstimulated and LPS/ox-LDL-challenged groups without kimchi, received the same apical DMSO concentration. For readability, the kimchi-derived soluble fractions are referred to as S-K and LMS-K throughout the manuscript.

### Cell Culture and Differentiation

Caco-2 cells were cultured in Dulbecco's Modified Eagle Medium (DMEM; WELGENE, Republic of Korea; Cat. No. LM001-05) supplemented with 10% (v/v) fetal bovine serum (FBS; WELGENE; Cat. No. S001-07), 1% non-essential amino acids (NEAA; WELGENE; Cat. No. LS005-01), and penicillin–streptomycin (100 U/mL; WELGENE; Cat. No. LS202-02). Cells were maintained at 37°C in a humidified incubator with 5% CO_2_, and the culture medium was replaced every 3 days. For differentiation, Caco-2 cells were seeded onto Transwell^®^ inserts and cultured for 21 days to establish a differentiated intestinal epithelial monolayer. THP-1 cells were maintained in RPMI-1640 medium (WELGENE; Cat. No. LM011-01) supplemented with 10% (v/v) FBS, and penicillin–streptomycin (100 U/mL) at 37°C in a humidified atmosphere containing 5% CO_2_. To induce differentiation into macrophages, THP-1 cells were treated with 1 μM PMA for 48 h. The cells were then washed with phosphate-buffered saline (PBS) and incubated in fresh RPMI-1640 medium without PMA for an additional 24 h before subsequent experiments.

### Preparation of Macrophage-Conditioned Medium

Differentiated THP-1 macrophages, prepared as described above, were left unstimulated or stimulated with LPS (500 ng/mL) alone or in combination with ox-LDL (20 μg/mL) for 24 h. Culture supernatants were then collected directly from the respective treatment cultures and centrifuged at 300 × g for 5 min to remove detached cells and cellular debris. The resulting conditioned medium (CM), comprising macrophage-derived products and residual components of the corresponding treatment medium, was used directly to treat Caco-2 cells for 24 h.

### Immunofluorescence Analysis

Immunofluorescence staining was performed in the CM-validation and Caco-2/THP-1 co-culture experiments. For CM validation, THP-1-derived macrophages were stained for total NF-κB p65 (Santa Cruz Biotechnology, USA; Cat. No. sc-8008; clone F-6; 1:100) and CD36 (Abcam, UK; Cat. No. ab252922; clone EPR22509-40; 1:200), whereas Caco-2 monolayers exposed to the corresponding CM were stained for total NF-κB p65 and E-cadherin (Cell Signaling Technology, USA; Cat. No. 3195; clone 24E10; 1:1,000). In the co-culture experiments, THP-1-derived macrophages were stained for total NF-κB p65 and PPARα (Invitrogen, USA; Cat. No. PA1-822A; rabbit polyclonal; 1:100), and Caco-2 monolayers were stained for total NF-κB p65, phosphorylated NF-κB p65 (Ser536) (Cell Signaling Technology; Cat. No. 3033; clone 93H1; 1:1,000), and E-cadherin. Supplementary analysis of co-cultured THP-1-derived macrophages included staining for total NF-κB p65 and phosphorylated NF-κB p65 (Ser536).

Following treatment, cells were washed twice with PBS, fixed with 4% paraformaldehyde for 30 min at 4°C, permeabilized with 0.1% Triton X-100 for 10 min, and blocked with 3% BSA for 1 h at room temperature. Cells were incubated overnight at 4°C with the appropriate primary antibodies at the dilutions specified above, followed by Alexa Fluor 488-conjugated goat anti-mouse IgG secondary antibody (Invitrogen, Thermo Fisher Scientific, USA; Cat. No. A-11001; 1:2,000) or Alexa Fluor 555-conjugated goat anti-rabbit IgG secondary antibody (Invitrogen, Thermo Fisher Scientific; Cat. No. A-21428; 1:2,000) for 1 h at room temperature. Nuclei were counterstained with DAPI, and images were acquired using an LSM 880 confocal microscope (Carl Zeiss) with identical acquisition settings across groups within each analysis.

Background-corrected mean fluorescence intensity (MFI) was quantified using ImageJ within cellular regions of interest (ROIs) defined using a consistent threshold across images for each target. Three fields were randomly selected per independent biological replicate, and quantification was performed with the investigator blinded to treatment groups. MFI values from the three fields were averaged to obtain one value per biological replicate, without normalization to a control group. Statistical analyses were based on three independent biological replicates (n = 3), with fields not treated as independent observations.

### Cell Viability Assay

Cell viability following S-K or LMS-K treatment was assessed in THP-1-derived macrophages and Caco-2 cells using the MTS assay. Cells were seeded in 96-well plates at 1 × 10^4^ cells/well and incubated overnight. For the MTS assay, concentrated stocks of S-K and LMS-K were prepared separately in DMSO at 200 mg/mL (kimchi-equivalent) and diluted in culture medium to achieve final kimchi-equivalent concentrations of 0.5, 1.0, 1.5, or 2.0 mg/mL. The final DMSO concentration was adjusted to 1% (v/v) across all groups, including the vehicle control containing no kimchi preparation (0 mg/mL). Following 24 h of treatment, MTS reagent was added to each well, and the plates were incubated for 2 h at 37°C. Absorbance was measured at 490 nm using a microplate reader (Tecan, Zurich, Switzerland), and cell viability was expressed as a percentage of the vehicle control.

### Caco-2/THP-1 Transwell Co-Culture and Treatment

Caco-2 cells were seeded at 1 × 10^5^ cells/well on 0.4-μm Transwell^®^ inserts and cultured for 21 days to establish differentiated intestinal epithelial monolayers, with medium replacement every 3 days. THP-1 cells were seeded at 1 × 10^6^ cells/well in 6-well plates and differentiated into macrophages as described above. Following macrophage differentiation and recovery, the inserts containing Caco-2 monolayers were transferred to the macrophage-containing wells to establish the co-culture system.

The apical compartment contained 1 mL of DMEM supplemented with 10% FBS, and the basolateral compartment contained 2 mL of RPMI-1640 supplemented with 10% FBS. S-K or LMS-K was added to the apical compartment at a kimchi-equivalent concentration of 1 mg/mL. The initial apical DMSO concentration was maintained at 1% (v/v) across all groups, including the unstimulated and challenged controls without kimchi. In the challenged groups, LPS (500 ng/mL) and ox-LDL (20 μg/mL) were added to the basolateral compartment only; the unstimulated control received neither stimulus.

Co-cultures were incubated for 24 h at 37°C in a humidified atmosphere containing 5% CO_2_. Caco-2 cells and THP-1 macrophages were then harvested separately from the inserts and lower wells, respectively, for subsequent analyses.

### RNA Isolation and Quantitative PCR

Total RNA was isolated using TRIzol reagent (Invitrogen, USA; Cat No. 15596018) according to the manufacturer’s instructions. RNA concentration and purity were evaluated by measuring absorbance at 260 and 280 nm using a NanoDrop 2000 spectrophotometer (Thermo Fisher Scientific). First-strand cDNA was synthesized from 1 μg of total RNA using a cDNA synthesis kit (Enzynomics, Republic of Korea; Cat No. EZ005S). Quantitative PCR was performed using SYBR Green chemistry on a CFX96 Touch Real-Time PCR Detection System (Bio-Rad, USA). Relative gene expression levels were calculated using the 2^−ΔΔCt^ method after normalization to *GAPDH*. Relative expression is presented as fold change relative to the unstimulated vehicle control. The primers purchased from Bioneer were as follows: human *RELA*, forward 5′-GCATCTCCCTGGTCACCA-3′ and reverse 5′-GGAAGGGGTTGTTGTTGGTC-3′; *PTGS2*, forward 5′-TGTGTTTGGAGTGGGTTTCAG-3′and reverse 5′-TAAGTGCGATTGTACCCGGA-3′; *TJP1*, forward 5′-TCAGAGTGGGGAAACGTCAA-3′ and reverse 5′-CCCACTTTTC CTTAGTTGCTGA-3′; *PPARA*, forward 5′-AGGTGGAGTCTGAGCACATG-3′ and reverse 5′-ACAAAGACGGGATGCTGGTA-3′; *NR1H3*, forward 5′-ACATATGTGGAAGCCCTGCA-3′ and reverse 5′-AGTTTCATTAGCATCCGTGGG-3′; *CD36*, forward 5′-ACATCAGGACTT GCATACAGAA-3′ and reverse 5′-TGTTCTTCCATCCAAGGCCT-3′; *OLR1*, forward 5′-GCCTGGGATTAGTAGTGACCA-3′ and reverse 5′-CAGATTCTGGTGGTGAAGTTCC-3′; *GAPDH*, forward 5′-GCACCGTCAAGGCTGAGAAC-3′ and reverse 5′-ATGGTGGTGAAGACGCCA GT-3′.

### Western Blot Analysis

Western blot analysis was performed to assess the expression of proteins associated with inflammation, lipid metabolism, and cholesterol homeostasis. Total protein (30 μg) was separated by sodium dodecyl sulfate-polyacrylamide gel electrophoresis (SDS-PAGE) and transferred onto nitrocellulose membranes (Invitrogen; Cat No. 88018). Membranes were blocked for 2 h at room temperature in PBS containing 0.1% Tween 20 (PBST) and 5% nonfat dry milk, followed by overnight incubation at 4°C with primary antibodies against LXRα/β (Santa Cruz; Cat. No. sc-377260; clone H-7; 1:1,000), PPARα (Invitrogen; Cat. No. PA1-822A; rabbit polyclonal; 1:1,000), CD36 (Abcam; Cat. No. ab252922; clone EPR22509-40; 1:1,000), LOX-1 (Santa Cruz; Cat. No. sc-66155; clone LOX19-22; 1:1,000), total NF-κB p65 (Santa Cruz; Cat. No. sc-8008; clone F-6; 1:1,000), COX-2 (Santa Cruz; Cat. No. sc-376861; clone H-3; 1:500), and GAPDH (Santa Cruz; Cat. No. sc-47724; clone 0411; 1:1,000). After three washes with PBST for 10 min each, membranes were incubated for 1 h at room temperature with horseradish peroxidase (HRP)-conjugated anti-mouse IgG (Cell Signaling Technology; Cat. No. 7076; 1:2,000) or anti-rabbit IgG (Cell Signaling Technology; Cat. No. 7074; 1:2,000) secondary antibodies.

Protein bands were detected using an enhanced chemiluminescence (ECL) reagent, and band intensities were quantified using ImageJ software. Target-protein band intensities were normalized to the corresponding GAPDH band intensity within each of three independent biological experiments (n = 3). The normalized values were used for quantitative analysis and are presented as individual data points with the mean ± SD. Representative blots are shown. Source images from the three biological experiments are provided in Supplementary Fig. S2. The LXR antibody recognizes both LXRα and LXRβ; the corresponding Western blot signal is therefore reported as LXRα/β immunoreactivity.

### FITC-Dextran Permeability Assay

Paracellular permeability of Caco-2 monolayers was evaluated using FITC-dextran. After treatment, the apical and basolateral compartments were washed with PBS and replaced with serum-free medium. FITC-dextran (4 kDa) was added to the apical compartment at a final concentration of 1 mg/mL, while serum-free medium was added to the basolateral compartment. After incubation at 37°C for 2 h, medium from the basolateral compartment was collected and transferred to a black 96-well plate. Fluorescence intensity was measured using a microplate reader at an excitation wavelength of 485 nm and an emission wavelength of 535 nm. Paracellular permeability was expressed as the relative fluorescence intensity normalized to the unstimulated vehicle control group, which was set to 1.0.

### Oil Red O Staining

Intracellular lipid accumulation was assessed by Oil Red O staining. After treatment, the cells were washed twice with PBS, fixed with 4% paraformaldehyde for 30 min at 4°C, and stained with a freshly prepared Oil Red O working solution for 30 min at room temperature. The cells were then washed thoroughly with distilled water, and representative images were captured using a Nikon Eclipse TS2 microscope (Nikon, Japan). The retained dye was eluted with 100% isopropanol, and the absorbance was measured at 520 nm using a microplate reader (Tecan, Switzerland). Intracellular lipid accumulation was expressed as a percentage of the LPS/ox-LDL-treated group, which was set to 100%.

### Experimental Replication and Statistical Analysis

All quantitative experiments included three independent biological replicates (n = 3), each performed using independently prepared cell cultures. Technical replicate wells, microscopic fields, and repeated instrument readings were averaged within each biological replicate and were not treated as independent observations. Individual data points represent independent biological replicates, and data are presented as individual values with the mean ± SD.

Statistical analyses were performed using GraphPad Prism 7 (GraphPad Software, USA). One-way analysis of variance (ANOVA) followed by Dunnett’s multiple-comparisons test compared each MTS treatment concentration with its vehicle control (Fig. 1).[Fig F2] For co-culture experiments (Figs. 3–7), the unstimulated vehicle control and each kimchi-treated group were compared with the LPS/ox-LDL-challenged vehicle control. No direct comparison between S-K and LMS-K was performed. Statistical significance was defined as *p* < 0.05.

## Results

### Selection of a Non-Cytotoxic Kimchi Concentration for Caco-2 Cells and THP-1-Derived Macrophages

To establish a non-cytotoxic concentration for subsequent co-culture experiments, cell viability was assessed in Caco-2 cells and THP-1-derived macrophages following treatment with S-K or LMS-K (Fig. 1A–1D). Neither S-K nor LMS-K affected cell viability at concentrations of 0.5 or 1.0 mg/mL in either cell type. In contrast, concentrations of 1.5 and 2.0 mg/mL reduced cell viability in both cell types. Therefore, 1.0 mg/mL was selected as the treatment concentration for the subsequent Caco-2/THP-1 co-culture experiments.

### Establishment and Characterization of the Compartmentalized Caco-2/THP-1 Co-Culture Model

The intestinal epithelium is exposed to luminal factors and soluble mediators released by underlying macrophages (Fig. 2A). To model this interaction, a compartmentalized Caco-2/THP-1 Transwell system was established (Fig. 2B). Differentiated Caco-2 monolayers were placed in the apical compartment, and THP-1-derived macrophages were cultured in the basolateral compartment. Kimchi preparations were applied apically, whereas macrophages were stimulated with LPS and ox-LDL.

Before initiating the co-culture experiments, we first characterized macrophage responses to inflammatory and lipid stress and examined the effects of the resulting conditioned media on Caco-2 monolayers. The conditioned media were collected directly from the macrophage stimulation cultures and therefore contained macrophage-derived soluble products together with residual LPS/ox-LDL. In THP-1-derived macrophages, LPS stimulation increased total NF-κB p65 and CD36 staining, while the addition of ox-LDL further enhanced both signals, with the most pronounced change observed for CD36 (Fig. 2C). Based on these findings, combined LPS/ox-LDL treatment was selected as the inflammatory and lipid-stress condition for the co-culture experiments. When applied to Caco-2 monolayers, CM from LPS-treated macrophage cultures increased total NF-κB p65 staining and altered the E-cadherin staining pattern. These changes were more apparent following exposure to CM from macrophages treated with both LPS and ox-LDL (Fig. 2D).

### Apical S-K and LMS-K Attenuate Epithelial Inflammatory Responses in the Caco-2/THP-1 Co-Culture Model

To assess epithelial inflammatory responses in the co-culture model, we first examined *RELA* and *PTGS2* mRNA expression in Caco-2 cells following apical S-K or LMS-K treatment under basolateral LPS/ox-LDL challenge. LPS/ox-LDL significantly increased *RELA* expression relative to the unstimulated co-culture control, whereas apical treatment with either kimchi preparation significantly reduced its expression relative to the LPS/ox-LDL-treated group (Fig. 3A). *PTGS2* expression followed a similar pattern (Fig. 3B).

We next examined whether these transcriptional responses were accompanied by changes in total and phosphorylated NF-κB p65 signals. Immunofluorescence analysis showed that LPS/ox-LDL challenge significantly increased the mean fluorescence intensities of both total and phosphorylated NF-κB p65, whereas apical treatment with either S-K or LMS-K significantly reduced both signals (Fig. 3C–3E). Taken together, these results show that apical treatment with either kimchi preparation attenuated epithelial inflammatory marker responses under basolateral LPS/ox-LDL challenge.

### Apical S-K and LMS-K Attenuate Epithelial Barrier-Associated Disruption in the Caco-2/THP-1 Co-Culture Model

To evaluate epithelial barrier integrity, we measured FITC-dextran passage across differentiated Caco-2 monolayers as a functional indicator of paracellular permeability and examined *TJP1* mRNA expression and E-cadherin staining as barrier-associated markers. Basolateral LPS/ox-LDL challenge increased FITC-dextran passage, whereas apical treatment with either S-K or LMS-K significantly reduced passage compared with the LPS/ox-LDL-treated group (Fig. 4A). LPS/ox-LDL challenge also reduced *TJP1* mRNA expression in Caco-2 cells. Apical LMS-K treatment significantly increased *TJP1* expression relative to the LPS/ox-LDL-treated group, whereas S-K treatment did not produce a significant change (Fig. 4B).

E-cadherin staining showed changes in both the distribution and intensity of the epithelial signal. Representative images showed a less continuous staining pattern along epithelial cell borders under LPS/ox-LDL-stimulated conditions (Fig. 4C). In parallel, mean E-cadherin fluorescence intensity was significantly higher in both the S-K- and LMS-K-treated groups than in the LPS/ox-LDL-treated group (Fig. 4D).

Together, the reduced FITC-dextran passage and increased E-cadherin signal indicate that apical S-K and LMS-K treatment attenuated epithelial barrier-associated disruption in the Caco-2/THP-1 co-culture model.

### Apical Treatment with S-K or LMS-K Is Associated with Reduced Inflammatory Responses in Co-Cultured THP-1-Derived Macrophages

To determine whether apical S-K or LMS-K treatment was accompanied by changes in basolateral macrophages, we examined inflammatory marker expression in co-cultured THP-1-derived macrophages. Compared with the unstimulated co-culture control, LPS/ox-LDL challenge significantly increased *PTGS2* and *RELA* mRNA expression. Apical treatment with either S-K or LMS-K significantly reduced both transcripts relative to the LPS/ox-LDL-treated group without kimchi treatment (Fig. 5A and 5B). Western blot quantification showed significantly higher GAPDH-normalized COX-2 and total NF-κB p65 protein levels following LPS/ox-LDL challenge and significantly lower levels in both kimchi-treated groups than in the LPS/ox-LDL-treated group (Fig. 5C–5E).

Immunofluorescence analysis further showed that LPS/ox-LDL challenge increased total NF-κB p65 fluorescence intensity and decreased PPARα fluorescence intensity. Apical treatment with either S-K or LMS-K significantly reduced total NF-κB p65 fluorescence and increased PPARα fluorescence relative to the LPS/ox-LDL-treated group (Fig. 5F–5H). Additional immunofluorescence analysis of phosphorylated NF-κB p65 (Ser536) showed a similar staining pattern, with stronger staining following LPS/ox-LDL challenge and lower staining in both kimchi-treated groups (Supplementary Fig. S1).

Together, these results indicate that apical exposure to either kimchi preparation was accompanied by a consistent reduction in inflammatory marker expression and NF-κB-associated signals, together with increased PPARα fluorescence, in basolateral macrophages within the compartmentalized co-culture system.

### Apical S-K and LMS-K Treatment Is Associated with Reduced Lipid Accumulation in Co-Cultured THP-1-Derived Macrophages

We next assessed lipid accumulation in the basolateral macrophage compartment. Compared with the unstimulated co-culture control, LPS/ox-LDL stimulation increased intracellular lipid accumulation in THP-1-derived macrophages, as shown by Oil Red O staining (Fig. 6A). Apical treatment with either S-K or LMS-K reduced the staining intensity. Quantification of the eluted Oil Red O dye showed significantly lower absorbance in both kimchi-treated groups than in the LPS/ox-LDL-treated group (Fig. 6B).

Together, these findings suggest that apical S-K and LMS-K treatment may reduce lipid loading in THP-1-derived macrophages within the compartmentalized epithelial–macrophage co-culture model.

### Apical S-K and LMS-K Modulate Lipid-Regulatory and ox-LDL Uptake-Related Markers in Co-Cultured THP-1-Derived Macrophages

To further characterize the molecular changes associated with reduced lipid accumulation, lipid-regulatory and ox-LDL uptake-related markers were examined in co-cultured THP-1-derived macrophages.

LPS/ox-LDL stimulation reduced *PPARA* and *NR1H3* mRNA expression relative to the unstimulated control. LMS-K treatment significantly increased the expression of both genes compared with the LPS/ox-LDL-treated group. S-K treatment significantly increased *NR1H3* expression, whereas *PPARA* did not increase significantly (Fig. 7A and 7B). Western blot quantification showed significantly lower GAPDH-normalized PPARα protein levels and LXRα/β immunoreactivity following LPS/ox-LDL stimulation. Apical treatment with either S-K or LMS-K significantly increased both measures relative to the LPS/ox-LDL-treated group (Fig. 7C–7E).

We next examined markers related to ox-LDL uptake. LPS/ox-LDL stimulation increased *CD36* and *OLR1* mRNA expression in THP-1-derived macrophages. Apical treatment with either S-K or LMS-K significantly reduced the expression of both genes compared with the LPS/ox-LDL-treated group (Fig. 7F and 7G). Western blot quantification showed significantly higher GAPDH-normalized CD36 and LOX-1 protein levels following LPS/ox-LDL stimulation and significantly lower levels in both kimchi-treated groups than in the LPS/ox-LDL-treated group (Fig. 7H–7J).

Together, these findings indicate that the reduced macrophage lipid accumulation observed in co-cultures receiving apical S-K or LMS-K treatment was accompanied by higher PPARα protein levels and LXRα/β immunoreactivity and lower expression of the ox-LDL uptake-related markers CD36 and LOX-1.

## Discussion

This study established a compartmentalized Caco-2/THP-1 Transwell model of inflammatory lipid stress and used it to evaluate the effects of apically administered S-K and LMS-K on epithelial and macrophage responses. Within this system, apical treatment with either preparation attenuated epithelial inflammatory responses and barrier-associated disruption and was accompanied by lower inflammatory marker expression and lipid accumulation in basolateral macrophages. The compartmentalized design allowed both sets of responses to be examined within the same experimental system while maintaining distinct sites of treatment and challenge.

In the epithelial compartment, apical treatment with either S-K or LMS-K was associated with lower *RELA* and *PTGS2* mRNA expression and reduced total and phosphorylated NF-κB p65 signals during basolateral LPS/ox-LDL challenge. Both preparations also reduced FITC-dextran passage, consistent with lower paracellular permeability. Among the barrier-associated markers, only LMS-K significantly increased *TJP1* mRNA expression relative to the LPS/ox-LDL-treated group, whereas both preparations increased mean E-cadherin fluorescence intensity. Consistent with this increase, the kimchi-treated monolayers showed a more continuous E-cadherin staining pattern along epithelial cell borders than the LPS/ox-LDL-treated group. Together, the effects of S-K and LMS-K across these inflammatory and barrier-related readouts support attenuation of epithelial barrier-associated disruption under inflammatory lipid stress [11, 35].

In the basolateral compartment, apical treatment with either S-K or LMS-K was associated with lower *PTGS2* and *RELA* mRNA expression and reduced COX-2 and total NF-κB p65 protein signals in THP-1-derived macrophages. Representative images from both kimchi-treated groups also showed lower phosphorylated NF-κB p65 (Ser536) staining than the LPS/ox-LDL-treated group, consistent with the directional change in the total NF-κB p65 signal and providing qualitative support for the inflammatory marker results. Both preparations reduced intracellular lipid accumulation, as assessed by Oil Red O staining. In the kimchi-treated groups, lower lipid accumulation was accompanied by higher PPARα protein levels and LXRα/β immunoreactivity and lower CD36 and LOX-1 expression. Inflammation and lipid metabolism are closely interconnected in macrophages [22, 23]. Ox-LDL uptake through CD36 and LOX-1 can promote intracellular lipid accumulation and inflammatory signaling, whereas PPARα and LXRα contribute to fatty-acid metabolism and cholesterol handling [22, 23, 27]. Collectively, these results show that apical treatment with either kimchi preparation was associated with a macrophage profile characterized by lower inflammatory marker expression and lipid accumulation, together with accompanying changes in lipid-regulatory and ox-LDL uptake-related markers.

The treatment preparation and compartmentalized design are important for interpreting these findings. The applied material was a 0.22-μm-filtered kimchi-derived soluble fraction rather than intact freeze-dried kimchi powder. Macrophage responses following apical treatment may therefore reflect epithelial-derived mediators, transepithelial movement of soluble kimchi components, or both. Conversely, epithelial responses may integrate macrophage-derived products with the direct effects of the basolateral LPS/ox-LDL challenge. The conditioned-medium analysis similarly reflects the composite soluble environment generated during macrophage stimulation. Thus, the model captures response patterns across both compartments but does not resolve the individual routes underlying these effects.

Three additional limitations should be considered. First, the Caco-2/THP-1 cell lines and defined LPS/ox-LDL challenge do not fully reproduce the cellular and physiological complexity of the intestinal mucosa, particularly macrophage heterogeneity, mucus production, microbial communities, and systemic influences. Second, the lipid-related measurements describe changes in lipid accumulation and associated markers but do not distinguish the relative contributions of ox-LDL uptake, intracellular storage, and cholesterol efflux. Third, the absence of a direct compositional comparison prevents attribution of the observed responses to a specific microorganism or metabolite. Moreover, because no direct statistical comparison between S-K and LMS-K was performed, the results do not establish the superiority of either preparation. Future studies using washed-macrophage conditioned medium, matched cell-free controls, and analysis of epithelial-derived mediators could clarify the routes operating between the two compartments. Lipid-flux measurements and detailed compositional profiling could further identify the relevant cellular processes and active components.

Taken together, apical S-K and LMS-K treatment attenuated epithelial inflammatory responses and barrier-associated disruption and was accompanied by lower inflammatory marker expression and lipid accumulation in basolateral macrophages under LPS/ox-LDL challenge. These findings support the use of the compartmentalized Caco-2/THP-1 model to evaluate epithelial and macrophage responses to apically applied kimchi-derived soluble components under inflammatory lipid stress.

## Supplemental Materials

Supplementary data for this paper are available on-line only at http://jmb.or.kr.



## Figures and Tables

**Fig. 1:**
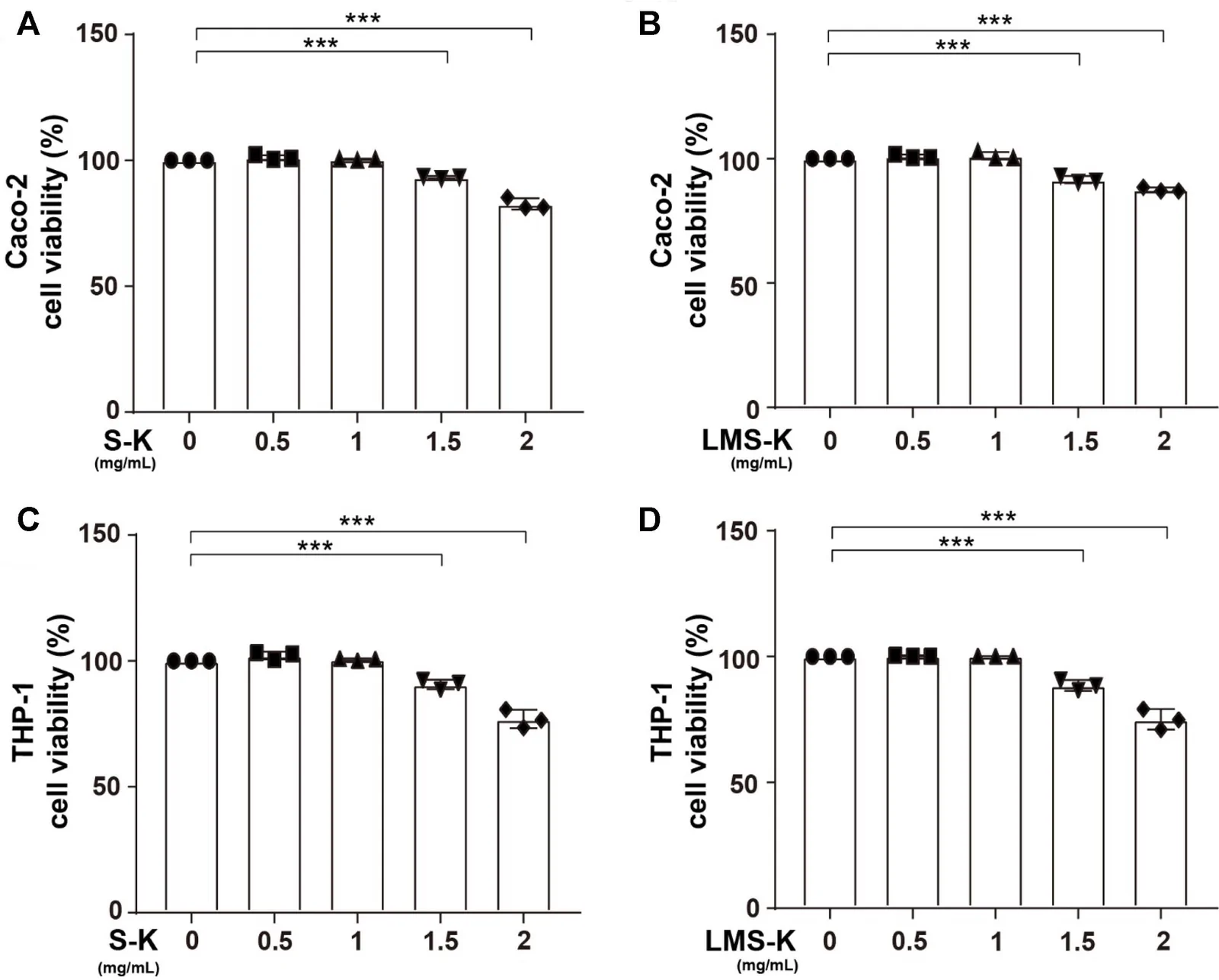
Effects of S-K and LMS-K on cell viability in Caco-2 cells and THP-1-derived macrophages. Caco-2 cells and THP-1-derived macrophages were treated with kimchi-derived soluble fractions from spontaneously fermented kimchi (S-K) or *Leuconostoc mesenteroides* KCKM0828 starter-fermented kimchi (LMS-K) at the indicated kimchi-equivalent concentrations for 24 h. The final DMSO concentration was matched at 1% (v/v) across all groups. Cell viability was assessed using the MTS assay. (**A, B**) Viability of Caco-2 cells treated with (**A**) S-K or (**B**) LMS-K. (**C, D**) Viability of THP-1-derived macrophages treated with (**C**) S-K or (**D**) LMS-K. Cell viability was expressed as a percentage of the corresponding vehicle control (0 mg/mL kimchi). Data are presented as mean ± SD, with each point representing one independent biological replicate (n = 3 per group). Statistical comparisons were performed using one-way ANOVA followed by Dunnett’s multiple-comparisons test against the corresponding vehicle control. ***p < 0.001.

**Fig. 2 F2:**
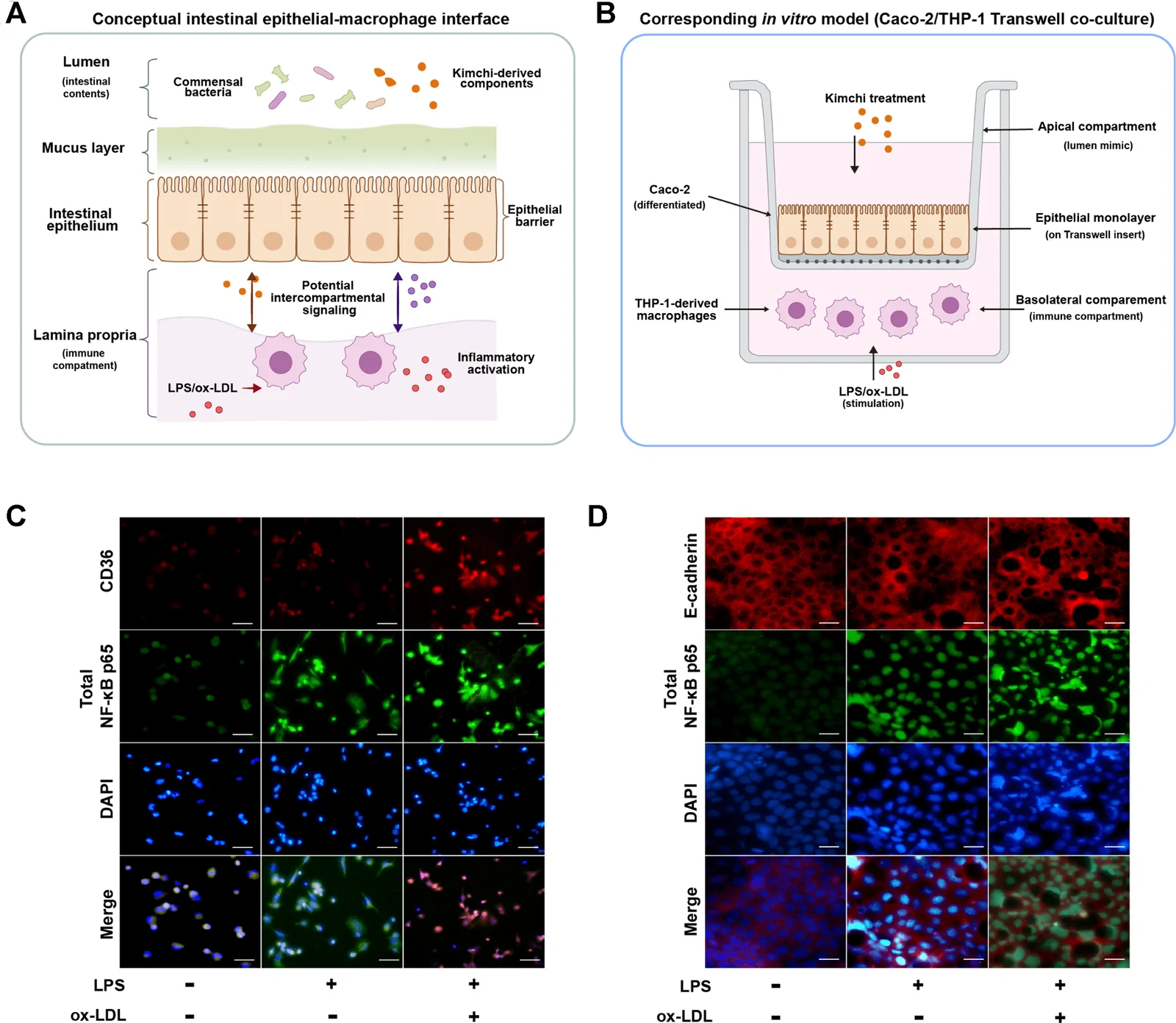
Experimental framework and cellular responses under inflammatory and lipid-stress conditions. (**A**) Schematic of the intestinal epithelial–macrophage interface and potential intercompartmental signaling. (**B**) Schematic of the Caco-2/THP-1 Transwell co-culture system. S-K or LMS-K was applied to the apical compartment, whereas LPS and ox-LDL were added to the basolateral compartment containing THP-1-derived macrophages. The basolateral surface of the Caco-2 monolayer was exposed to the lower-chamber medium through the porous membrane. (**C**) Representative immunofluorescence images of THP-1-derived macrophages left unstimulated or treated with LPS alone or in combination with ox-LDL, stained for CD36 (red) and total NF-κB p65 (green). (**D**) Representative immunofluorescence images of Caco-2 monolayers treated with conditioned medium collected directly from the corresponding macrophage cultures, comprising macrophage-derived products and residual components of the treatment medium. Cells were stained for E-cadherin (red) and total NF-κB p65 (green). Nuclei were counterstained with DAPI (blue). Images are representative of three independent biological experiments. Scale bars, 100 μm.

**Fig. 3:**
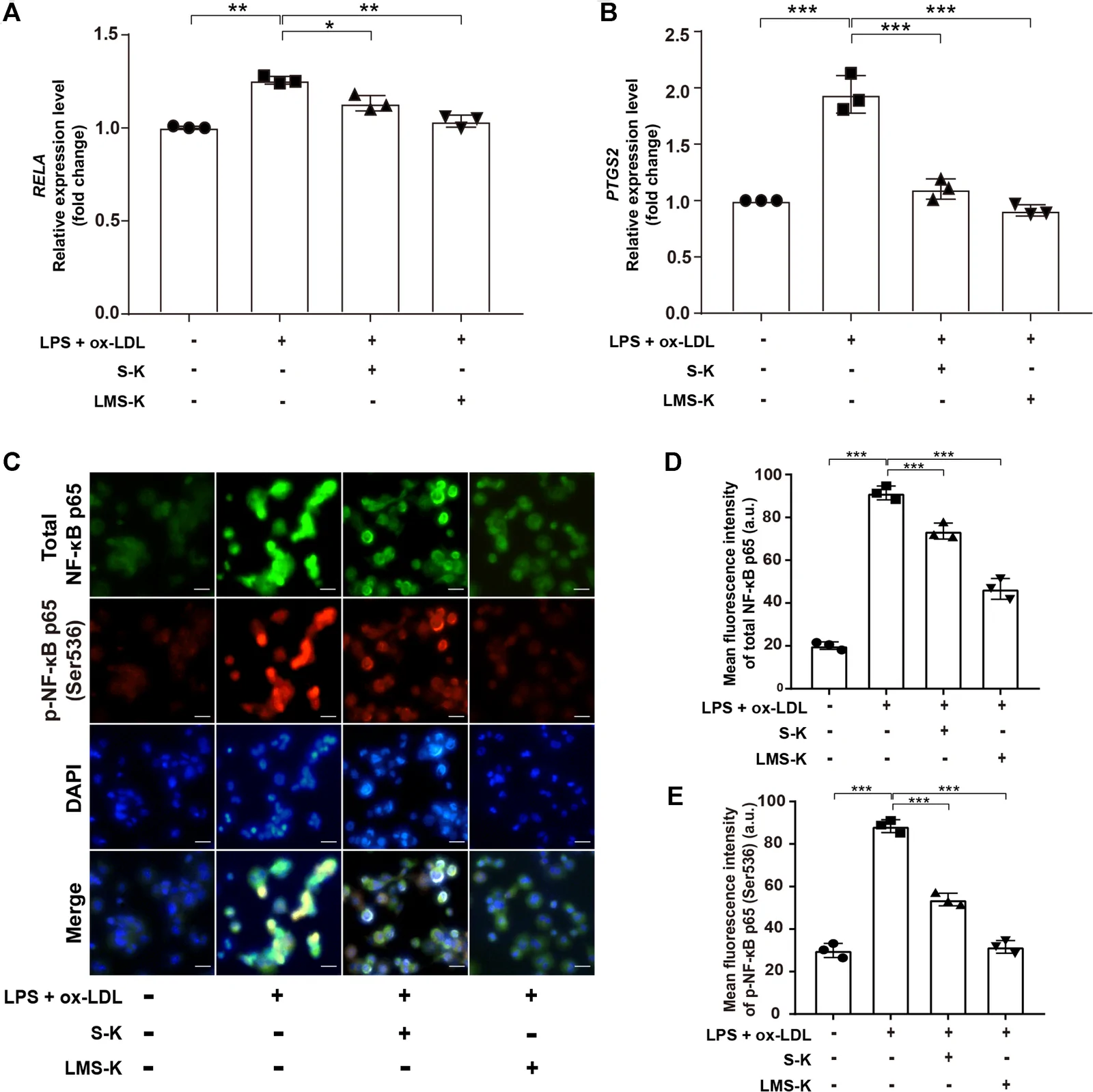
Effects of apical S-K and LMS-K treatment on inflammatory responses in co-cultured Caco-2 cells. (**A, B**) Relative mRNA expression of (**A**) *RELA* and (**B**) *PTGS2* in Caco-2 cells. (**C**) Representative immunofluorescence images of total NF-κB p65 (green), phosphorylated NF-κB p65 (Ser536) (red), and DAPI-stained nuclei (blue), with merged images shown in the bottom row. Scale bars, 100 μm. (**D, E**) Quantification of mean fluorescence intensity for (**D**) total NF-κB p65 and (**E**) phosphorylated NF-κB p65 (Ser536). Images are representative of three independent biological experiments. Quantitative data are presented as mean ± SD, with each point representing one independent biological replicate (n = 3 per group). Statistical comparisons were performed using one-way ANOVA followed by Dunnett’s multiple-comparisons test against the LPS/ox-LDL-challenged vehicle control. Brackets indicate the compared groups. *p < 0.05, **p < 0.01, and ***p < 0.001.

**Fig. 4:**
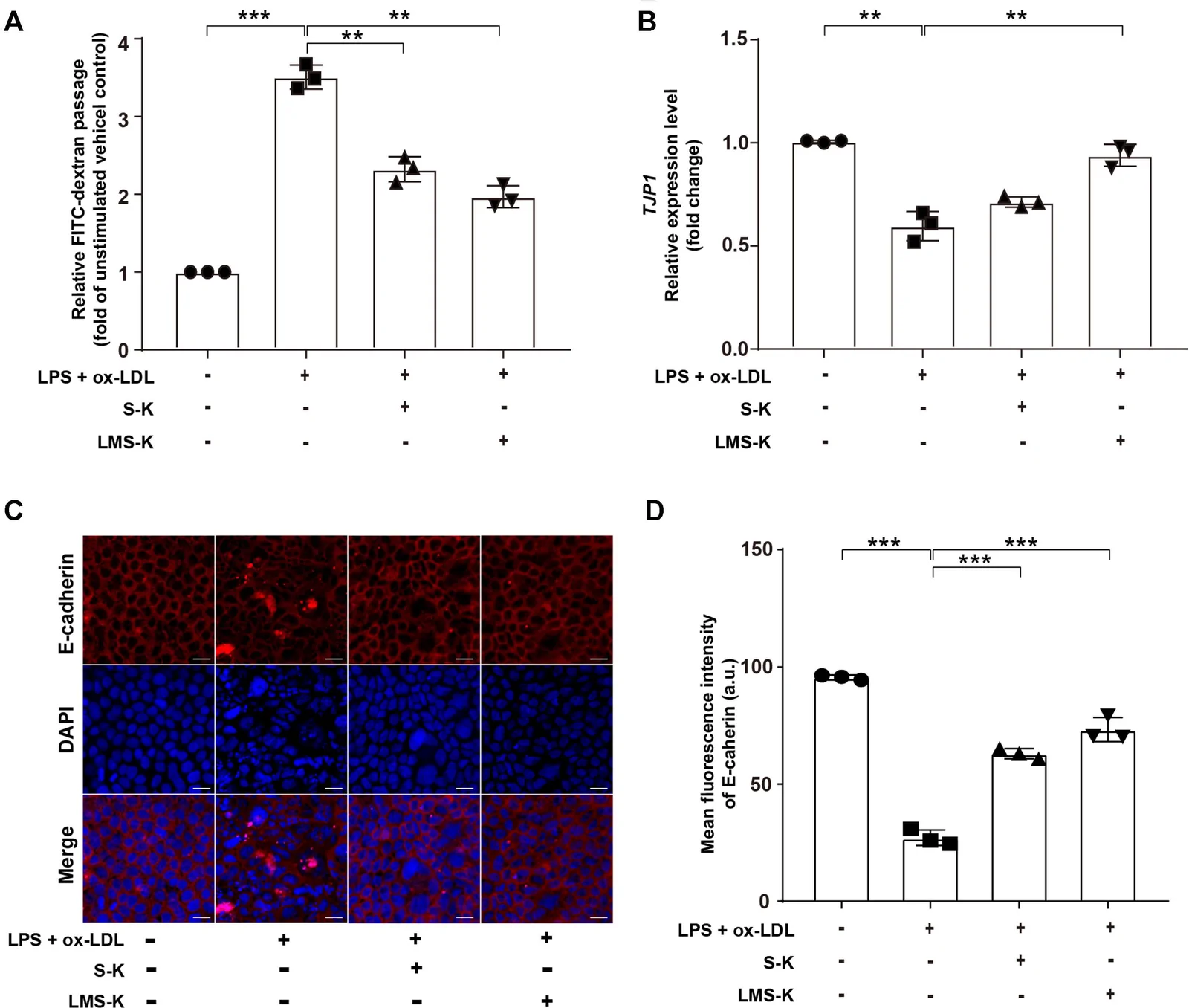
Effects of apical S-K and LMS-K treatment on epithelial barrier-related outcomes in the Caco-2/THP-1 co-culture system. (**A**) Relative FITC-dextran passage across differentiated Caco-2 monolayers into the basolateral compartment, expressed relative to the unstimulated vehicle control set to 1.0. (**B**) Relative *TJP1* mRNA expression in Caco-2 cells. (**C**) Representative immunofluorescence images of E-cadherin (red) and DAPI-stained nuclei (blue) in Caco-2 monolayers. Scale bar, 100 μm. (**D**) Quantification of mean E-cadherin fluorescence intensity. Images are representative of three independent biological experiments. Quantitative data are presented as mean ± SD, with each point representing one independent biological replicate (n = 3 per group). Statistical comparisons were performed using one-way ANOVA followed by Dunnett’s multiple-comparisons test against the LPS/ox-LDL-challenged vehicle control. Brackets indicate the compared groups. **p < 0.01 and ***p < 0.001.

**Fig. 5:**
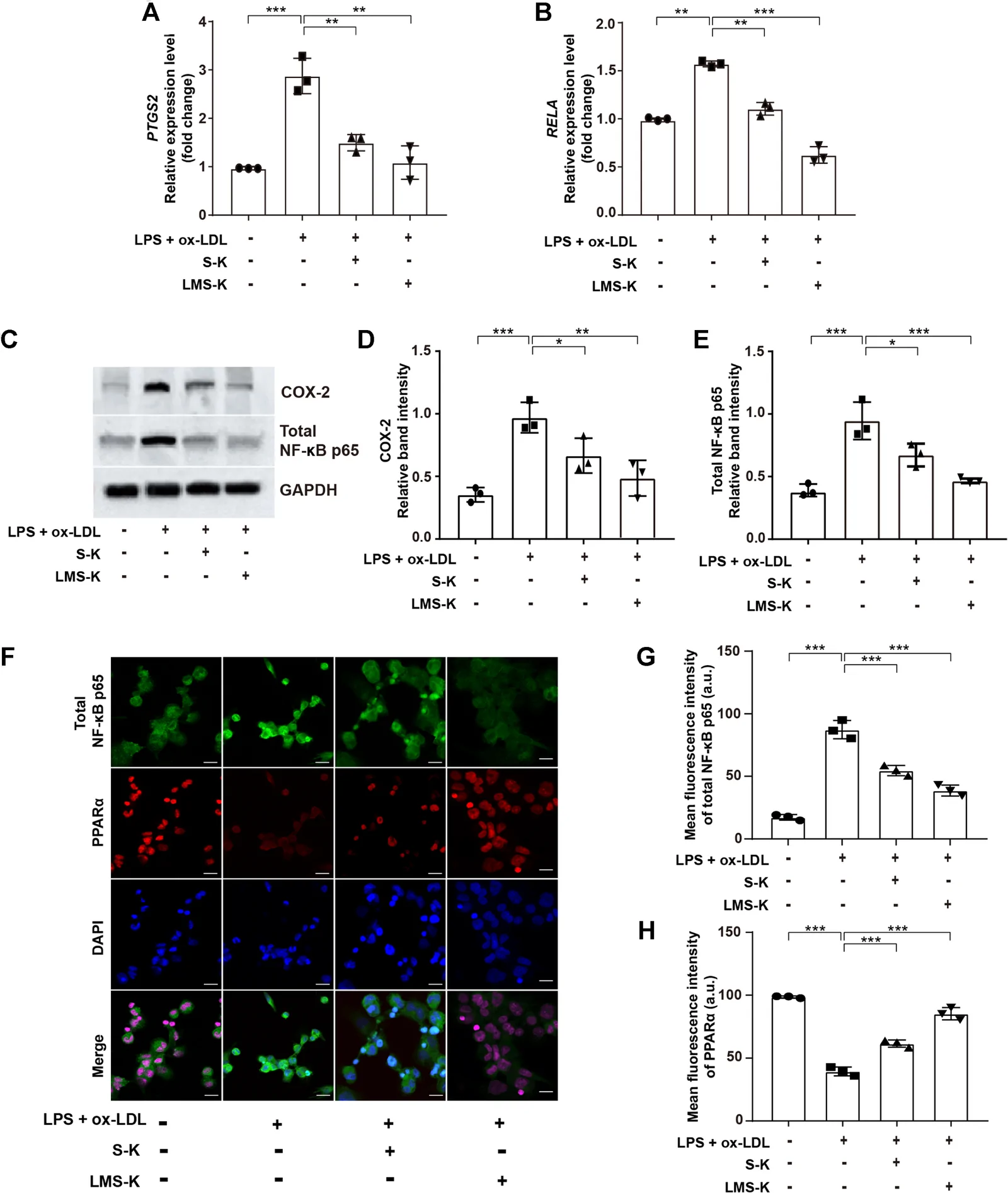
Effects of apical S-K and LMS-K treatment on inflammatory markers and PPARα in co-cultured THP-1-derived macrophages. (**A, B**) Relative mRNA expression of (**A**) *PTGS2* and (**B**) *RELA* in THP-1-derived macrophages. (**C**) Representative Western blots of COX-2 and total NF-κB p65, with GAPDH as the loading control. (**D, E**) Relative band intensities of (**D**) COX-2 and (**E**) total NF-κB p65 normalized to GAPDH. (**F**) Representative immunofluorescence images of total NF-κB p65 (green), PPARα (red), and DAPI-stained nuclei (blue). Scale bar, 100 μm. (**G, H**) Quantification of mean fluorescence intensity for (**G**) total NF-κB p65 and (**H**) PPARα. Blots and images are representative of three independent biological experiments. Quantitative data are presented as mean ± SD, with each point representing one independent biological replicate (n = 3 per group). Statistical comparisons were performed using one-way ANOVA followed by Dunnett’s multiple-comparisons test against the LPS/ox-LDL-challenged vehicle control. Brackets indicate the compared groups. *p < 0.05, **p < 0.01, and ***p < 0.001. Western blot source images are provided in Supplementary Fig. S2.

**Fig. 6:**
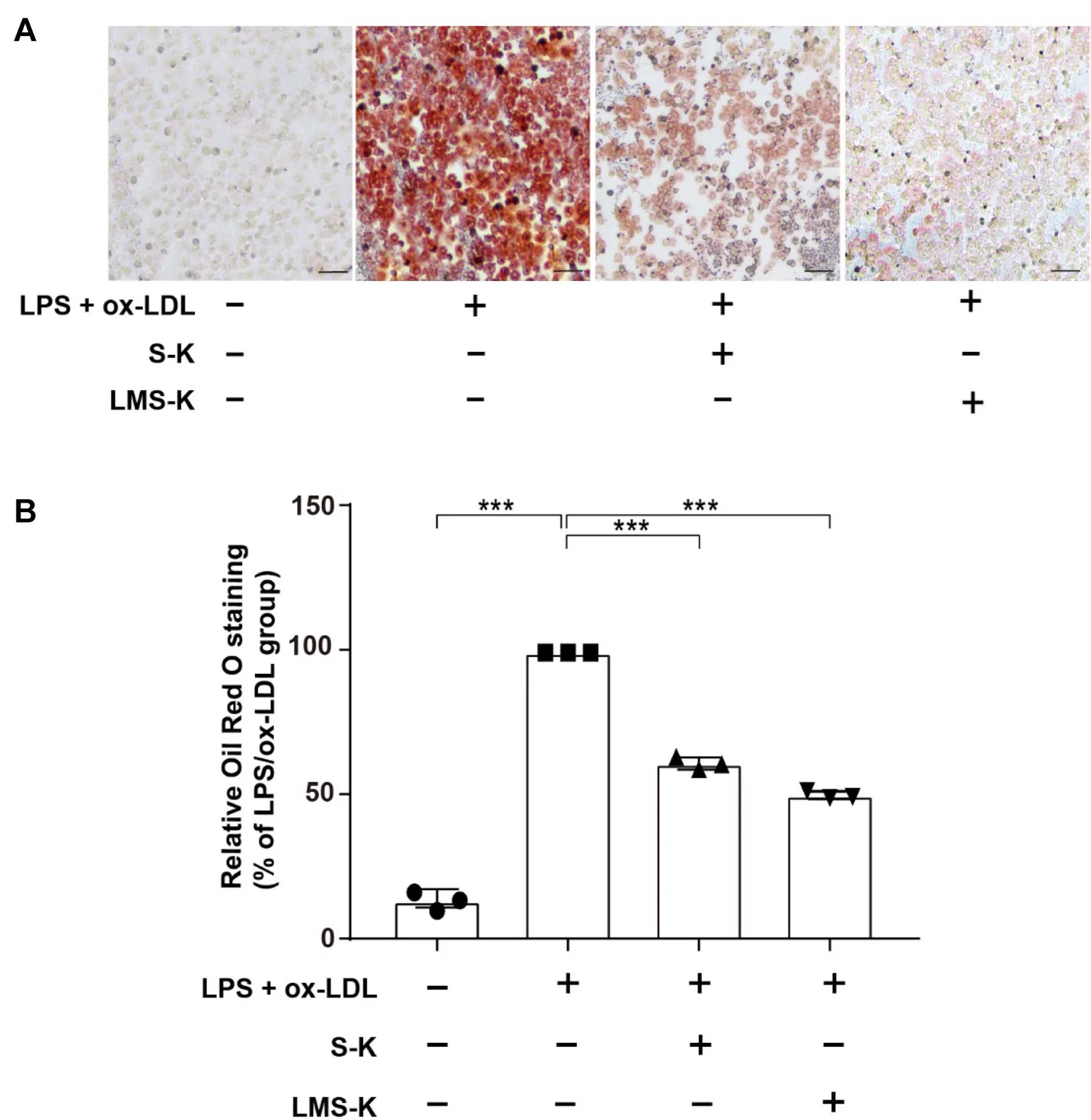
Effects of apical S-K and LMS-K treatment on lipid accumulation in co-cultured THP-1-derived macrophages. (**A**) Representative images of Oil Red O-stained THP-1-derived macrophages. Scale bar, 100 μm. (**B**) Quantification of Oil Red O staining based on the absorbance of eluted dye at 520 nm, expressed as a percentage of the LPS/ox-LDL-challenged vehicle control set to 100%. Images are representative of three independent biological experiments. Quantitative data are presented as mean ± SD, with each point representing one independent biological replicate (n = 3 per group). Statistical comparisons were performed using one-way ANOVA followed by Dunnett’s multiple-comparisons test against the LPS/ox-LDL-challenged vehicle control. Brackets indicate the compared groups. ***p < 0.001.

**Fig. 7:**
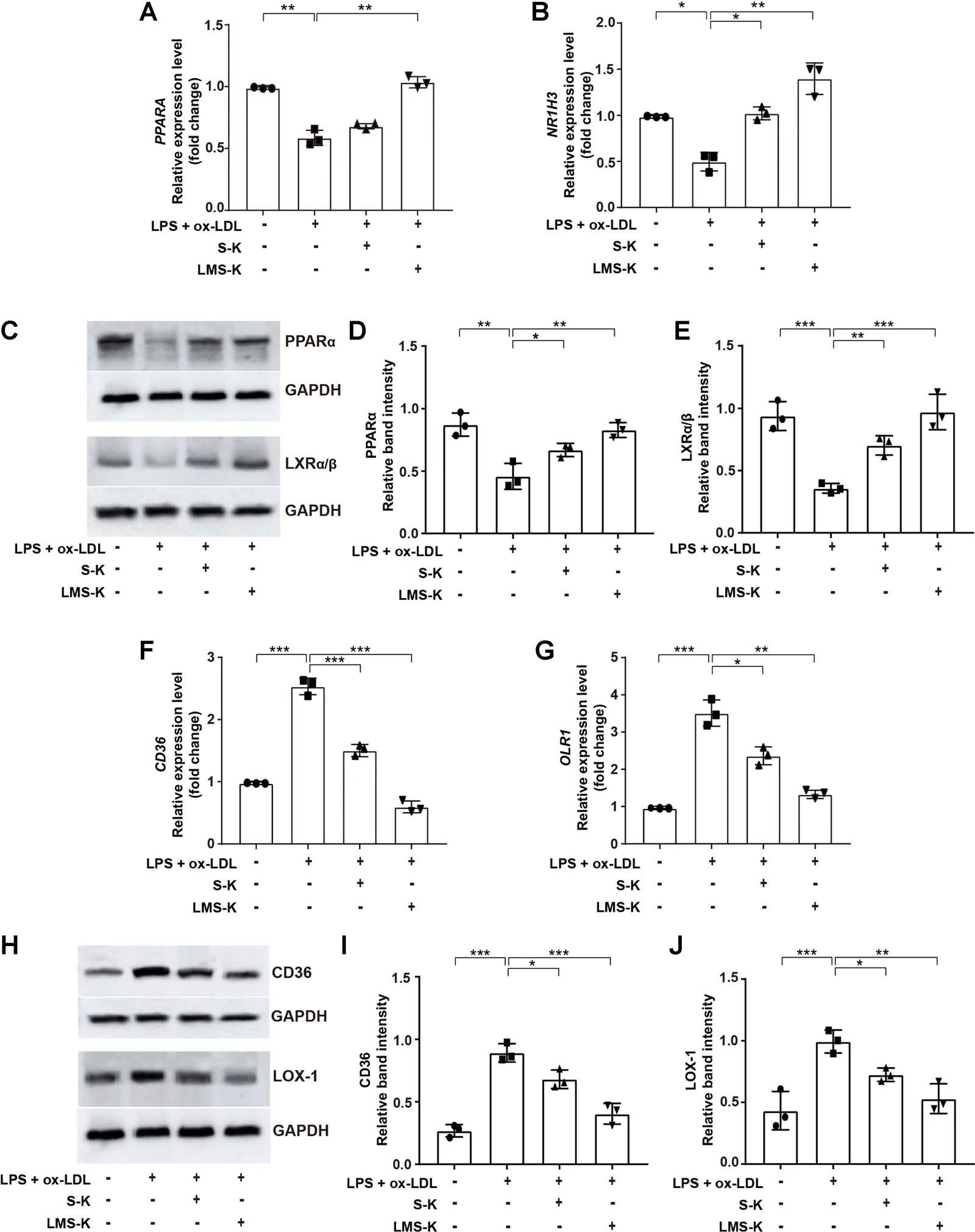
Effects of apical S-K and LMS-K treatment on lipid-regulatory markers and ox-LDL uptake receptors in co-cultured THP-1-derived macrophages. (**A, B**) Relative mRNA expression of (**A**) *PPARA* and (**B**) *NR1H3* in THP-1-derived macrophages. (**C**) Representative Western blots of PPARα and LXRα/β, with GAPDH as the loading control. (**D, E**) Relative band intensities of (**D**) PPARα and (**E**) LXRα/β normalized to GAPDH. (**F, G**) Relative mRNA expression of the ox-LDL uptake receptor genes (**F**) *CD36* and (**G**) *OLR1*. (**H**) Representative Western blots of CD36 and LOX-1, with GAPDH as the loading control. (**I, J**) Relative band intensities of (**I**) CD36 and (**J**) LOX-1 normalized to GAPDH. Blots are representative of three independent biological experiments. Quantitative data are presented as mean ± SD, with each point representing one independent biological replicate (n = 3 per group). Statistical comparisons were performed using one-way ANOVA followed by Dunnett’s multiple-comparisons test against the LPS/ox-LDL-challenged vehicle control. Brackets indicate the compared groups. *p < 0.05, **p < 0.01, and ***p < 0.001. Western blot source images are provided in Supplementary Fig. S2. LXRα/β denotes immunoreactivity detected with an antibody recognizing both isoforms.
