# Supplementary material for: Kimchi Modulates Epithelial–Macrophage Responses in a Caco-2/THP-1 Co-Culture Model of Inflammatory Lipid Stress
Source: J Microbiol Biotechnol. 2026 Oct 15;36:e2608002. doi: 10.4014/jmb.2608.08002 (PMC13725141; doi:10.4014/jmb.2608.08002)
Supplement: Supplementary file 1 [file jmb-36-e2608002-supple.pdf]

J. Microbiol. Biotechnol. 2026. 36: e2608002
https://doi.org/10.4014/jmb.2608.08002

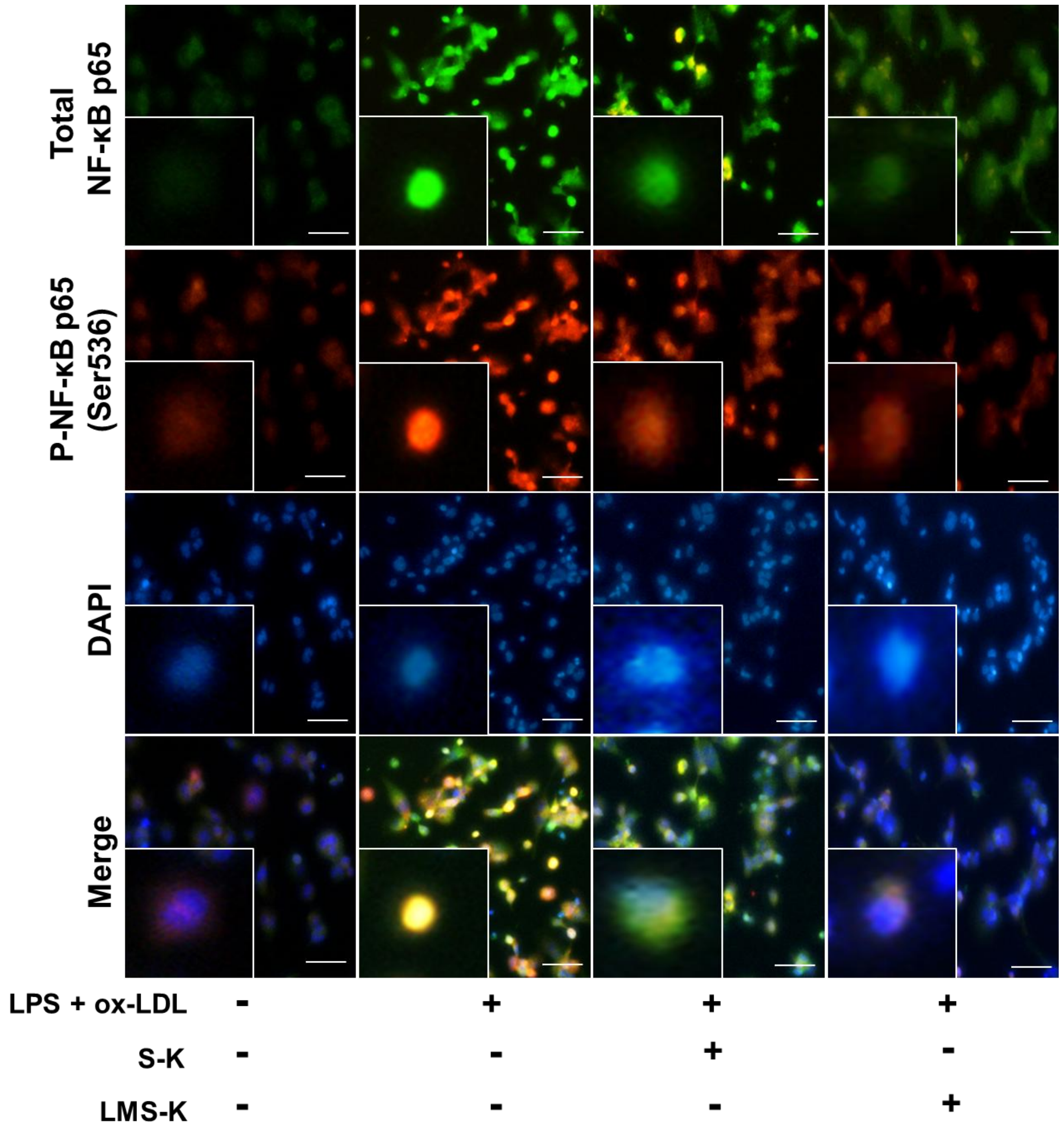

Fig. S1. Immunofluorescence staining of total and phosphorylated NF-κB p65 in co-cultured THP-1-derived macrophages.

THP-1-derived macrophages in the basolateral compartment were left unstimulated or challenged with LPS and ox-LDL, with or without apical S-K or LMS-K treatment. Representative immunofluorescence images show total NF-κB p65 (green), phosphorylated NF-κB p65 (Ser536) (red), and DAPI-stained nuclei (blue). Merged images are shown in the bottom row, with enlarged views of representative cells in the insets. Images were acquired using identical settings across groups and are representative of three independent biological experiments (n = 3). Scale bars in the main image panels, 100 μm.

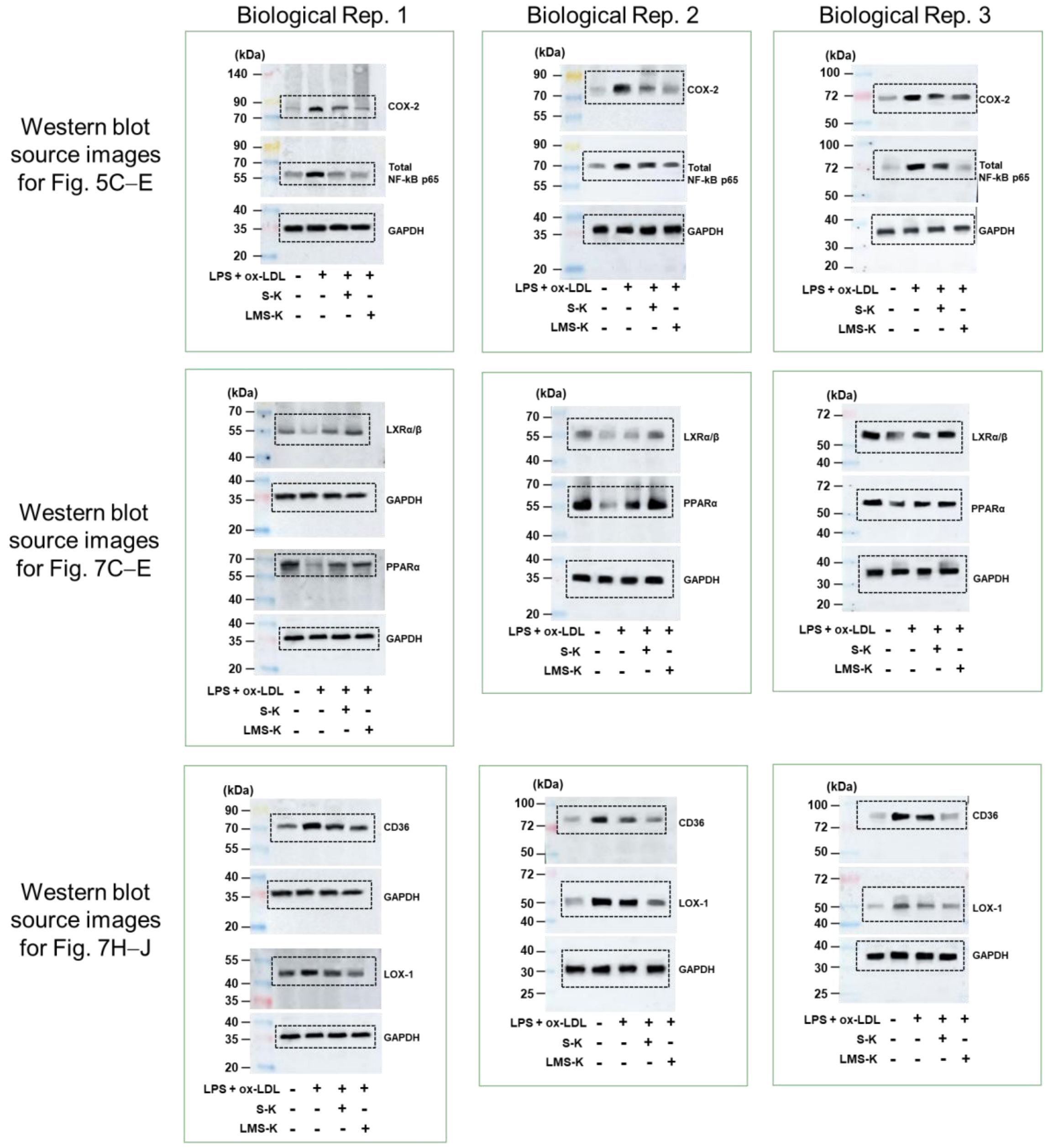

Fig. S2. Western blot source images from three independent biological experiments.

Source images correspond to the Western blot analyses presented in Fig. 5C–E (top row), Fig. 7C–E (middle row), and Fig. 7H–J (bottom row). Each column represents one independent biological experiment. Protein targets, molecular-mass markers (kDa), and lane treatment conditions are indicated. Dashed rectangles highlight the indicated band regions.
